# Redox-Dependent Modulation of Cardiac Mitochondrial F_1_F_O_-ATPase and Respiratory Function by N-Acetylcysteine and Sodium Ascorbate

**DOI:** 10.3390/biology15161360

**Published:** 2026-08-10

**Authors:** Antonia Cugliari, Cristina Algieri, Patrycja A. Glogowski, Fabiana Trombetti, Silvia Buscaroli, Micaela Fabbri, Ettore Federici, Salvatore Nesci

**Affiliations:** 1 Department of Veterinary Medical Sciences, University of Bologna, 40064 Ozzano Emilia, Italy; antonia.cugliari2@unibo.it (A.C.); fabiana.trombetti@unibo.it (F.T.); micaela.fabbri@unibo.it (M.F.); 2 Department of Life Sciences, Health and Health Professions, Link University, 00118 Rome, Italy; 3 Cardiovascular Medicine Unit, Heart, Chest and Vascular Department, IRCCS Azienda Ospedaliero-Universitaria di Bologna, 40138 Bologna, Italy; patrycja.glogowski2@unibo.it; 4 Mitochon Srl, 40064 San Lazzaro di Savena, Italy; silvia.buscaroli@mitochon.it (S.B.); ettore.federici@mitochon.it (E.F.)

**Keywords:** sodium ascorbate, N-acetylcysteine, antioxidants, thiol redox state, mitochondrial bioenergetic

## Abstract

Oxidative stress is closely associated with mitochondrial dysfunction and contributes to the development of several human diseases. Among antioxidant compounds, ASC and NAC are widely used for their cytoprotective and redox-regulating properties; however, their direct effects on specific aspects of mitochondrial bioenergetics are only partially characterized. In the present study, we investigated the effects of ASC and NAC, individually and combined, on Mg^2+^-dependent F_1_F_O_-ATPase hydrolysis, mitochondrial free thiol content and respiration in isolated swine heart mitochondria. ASC significantly stimulated F_1_F_O_-ATPase activity in a concentration-dependent manner, whereas kinetic analysis indicated a mixed uncompetitive activation mechanism. In contrast, NAC alone did not significantly affect F_1_F_O_-ATPase activity but abolished the stimulatory effect of ASC when the two compounds were combined. Both ASC and NAC increased mitochondrial free thiol content, although no change was observed under combined treatment conditions. Mitochondrial oxygen consumption analysis revealed substrate-dependent effects of the two antioxidants on electron transport. Overall, ASC and NAC exerted distinct direct actions on isolated mitochondria, and their combination did not produce additive or synergistic effects. These findings provide new insights into the direct modulation of mitochondrial function by antioxidant compounds and may contribute to expanding understanding of their potential therapeutic and dietary supplement applications, particularly when combined.

## Simple Summary

Antioxidant supplements are widely used to protect cells from oxidative stress. The aim of this study was to investigate the effects of two commonly used antioxidant compounds, sodium ascorbate (ASC) and N-acetylcysteine (NAC), on isolated swine heart mitochondria. The individual and combined effects of ASC and NAC on mitochondrial F_1_F_O_-ATPase activity, mitochondrial free thiol content and respiration were evaluated. ASC stimulated F_1_F_O_-ATPase hydrolytic activity, whereas NAC alone had no significant effect. However, under combined treatment conditions, NAC abolished the stimulatory effect of ASC. Furthermore, both antioxidants individually increased mitochondrial free thiol content, but no changes were observed with the combined treatment. In addition, ASC and NAC differentially affected mitochondrial oxygen consumption depending on the respiratory substrate, indicating their distinct direct actions on mitochondrial bioenergetics. These findings demonstrate that antioxidants with similar redox properties directly influence mitochondrial function through different mechanisms and highlight the importance of evaluating antioxidant combinations before their potential therapeutic use or application as nutritional supplement formulations.

## 1. Introduction

Mitochondria play a pivotal role in cellular energy metabolism by generating adenosine triphosphate (ATP) through oxidative phosphorylation (OXPHOS), a process driven by the electron transport chain (ETC) [1]. In particular, the energy released during electron transport among complexes I-IV is used to pump protons across the inner mitochondrial membrane, generating an electrochemical gradient that drives ATP synthesis by the F_1_F_O_-ATPase (complex V) [2]. Furthermore, mitochondria have significant roles in calcium balance, programmed cell death and production of reactive oxygen species (ROS) [3]. Under physiological conditions, ROS act as important signalling molecules involved in the regulation of cell proliferation, differentiation, and adaptive stress responses. However, excessive ROS production or impaired antioxidant defences result in oxidative stress [1,4]. In this regard, mitochondria are known as the major intracellular source of ROS formed during oxidative phosphorylation [5]. As a consequence, and because their mitochondrial DNA (mtDNA) is not protected by histones, their genetic material is more vulnerable to damage, which can lead to mitochondrial dysfunction. In addition, oxidative stress leads to oxidative damage of proteins and lipids, thereby compromising mitochondrial respiration and ATP production [6]. Growing evidence has implicated oxidative stress and mitochondrial dysfunction in the pathogenesis of numerous disorders, including neurodegenerative and cardiovascular diseases, metabolic disorders, cancer, and age-related conditions [7]. Consequently, strategies aimed at preserving mitochondrial function and redox homeostasis attract considerable interest as potential therapeutic approaches.

In this context, in the present study, we evaluated the effect of two well-known antioxidant compounds, sodium ascorbate (ASC) and N-acetyl-cysteine (NAC), on mitochondrial bioenergetics.

Sodium ascorbate, a sodium salt and a pH–neutral form of vitamin C, is known for its antioxidant and cytoprotective effects. Vitamin C is an essential nutrient that humans need to obtain through the diet because, unlike most mammals, we are unable to produce it from glucose [8]. It plays several vital roles in living organisms, acting as a cofactor for numerous enzymatic reactions [9]. It possesses the ability to remove superoxide anions and prevent the formation of peroxynitrite as well as improve vascular endothelial function [10]. For this reason, vitamin C supplementation was evaluated to reduce the overproduction of reactive oxygen species (ROS) and protect mitochondrial function [11]. Although the antioxidant benefits of sodium ascorbate have been extensively investigated in cellular and in vivo models, comparatively fewer studies have examined its direct effects on isolated heart mitochondria and also in combination with another relevant antioxidant compound, NAC.

N-acetylcysteine is an acetylated derivative of the amino acid L-cysteine and a precursor of glutathione (GSH), one of the major intracellular antioxidants and key regulator of cellular redox balance [12]. By replenishing intracellular GSH levels and preserving the sulfhydryl groups (-SH) of proteins in their reduced state, NAC protects against oxidative stress-induced damage, and owing to these properties, it garnered significant attention as a therapeutic antioxidant and anti-inflammatory agent [13] and is extensively included in various dietary supplements. Clinically, NAC is widely used for the treatment of acetaminophen overdose (paracetamol) and more recently as a mucolytic agent in respiratory diseases [12]. In addition, although NAC is not found in natural sources, it is commonly available as a nutritional supplement in many countries [12]. Similarly to ASC, NAC was widely investigated for its antioxidant properties and therapeutic potential in a range of diseases connected to oxidative stress, in particular neurodegenerative and cardiovascular diseases, cancer and psychiatric illnesses [14]. Several studies have also demonstrated that NAC can preserve mitochondrial function [15,16,17]. However, similarly to ASC, its direct effects on some aspects of mitochondrial bioenergetics, particularly on F_1_F_O_-ATPase activity, have not yet been explored; additionally, its combination with ASC may provide additive or synergistic effects on mitochondrial functionality.

To this end, the present study evaluated the effect of ASC and NAC on the hydrolytic activity of F_1_F_O_-ATPase, mitochondrial free thiol content and mitochondrial respiration, both individually and in combination.

## 2. Materials and Methods

### 2.1. Chemicals

Oligomycin (a mixture of oligomycins A, B and C), antimycin A, and succinate were purchased from Vinci-Biochem (Vinci, Italy). Na_2_ATP, rotenone, 5,5′-Dithio-bis-(2-nitrobenzoic acid) (DTNB), Nicotinamide Adenine Dinucleotide Hydride (NADH), sodium ascorbate (ASC) and N-Acetyl-L-cysteine (NAC) were obtained from Sigma–Aldrich (Milan, Italy). Quartz double distilled water was used for all reagent solutions.

### 2.2. Preparation of the Mitochondrial Fractions

Mitochondria were isolated from fresh swine hearts (Sus scrofa domesticus) obtained from a local abattoir and transported to the laboratory within 2 h in ice buckets at 0–4 °C. Visible fat and residual blood were removed as much as possible, and approximately 30–40 g of ventricular heart tissue was washed with ice-cold medium A (0.25 M sucrose, 10 mM Tris, pH 7.4 with HCl) and finely chopped into small pieces with scissors. Each preparation was made from one heart. Then, tissues were homogenized in medium B (0.25 M sucrose, 10 mM Tris, 1 mM EDTA, 0.5 mg/mL BSA, pH 7.4 with HCl) using a ratio of 10 mL of medium B per 1 g of fresh tissue. Samples were first disrupted with an homogenizer and subsequently homogenized using a manual teflon pestle homogenizer operated at 650 rpm with 3 gentle up-and-down strokes. The mitochondrial fractions were isolated by stepwise centrifugation. Briefly, the homogenate was centrifuged at 1000× *g* for 5 min, then the pellet was resuspended, homogenized once more under identical conditions, and centrifuged again at 1000× *g* for 5 min. The combined supernatants were filtered through four layers of cotton gauze and centrifuged at 10,500× *g* for 10 min to sediment crude mitochondria. The mitochondrial pellet was resuspended in medium A followed by a second centrifugation at 10,500× *g* for 10 min. The final pellet was gently resuspended in a small volume of medium A using a teflon pestle homogenizer to obtain a protein concentration of approximately 30 mg/mL. All isolation steps were performed at 0–4 °C to preserve mitochondrial integrity. The mitochondrial protein concentration was determined using the Bradford colorimetric assay with BSA as the calibration standard [18]. Purified mitochondrial preparations were stored in liquid nitrogen until the evaluation of F_1_F_O_-ATPase.

### 2.3. F-ATPase Hydrolytic Activity Assay

Frozen mitochondrial preparations were rapidly thawed immediately before use and employed for the determination of Mg^2+^-dependent F_1_F_O_-ATPase hydrolytic activity. ATP hydrolysis was measured in a final reaction volume of 1 mL under previously optimized assay conditions, consisting of 0.15 mg of mitochondrial protein suspended in 75 mM ethanolamine–HCl buffer (pH 9.0), containing 6.0 mM Na_2_ATP and 2.0 mM MgCl_2_. The reaction mixtures were pre-incubated for 5 min at 37 °C, after which the reaction was initiated by the addition of Na_2_ATP. The samples were then centrifuged at 2000× *g* for 15 min, and the amount of inorganic phosphate (Pi) released into the supernatant was determined spectrophotometrically according to the method previously described [19]. The released Pi was used as an indirect index of mitochondrial F_1_F_O_-ATPase activity. According to the method employed, to determine the oligomycin-sensitive ATPase activity, parallel reaction mixtures containing oligomycin were included in each experiment. Oligomycin was added directly to the reaction medium (1 μL of a 3 mg/mL stock solution prepared in dimethyl sulfoxide) before ATP addition to inhibit the F_1_F_O_-ATPase specifically through blockade of the F_O_ sector [20]. To test the effect of ASC on the F_1_F_O_-ATPase activity, aqueous dilutions of ASC at different standard concentrations were prepared immediately before each experimental set. Small aliquots of these solutions were added to the reaction system. The same was carried out for NAC but with one difference; in order to maintain the optimal pH, NAC dilutions were instead prepared in 75 mM ethanolamine–HCl buffer at pH 11.0 starting from a stock solution (250 mM) solubilized in 200 mM ethanolamine. Control tubes contained the same final volume of compound dilutions, adjusted with the reaction buffer.

In all experiments, the F_1_F_O_-ATPase activity was measured by subtracting, from the Pi hydrolyzed by total ATPase activity, the Pi hydrolyzed in the presence of oligomycin. In all experiments, the F-ATPase activity was expressed as µmol Pi∙mg protein^−1^ min^−1^.

### 2.4. Kinetic Study

To calculate kinetic parameters (*V_max_* and *K_m_*), the enzyme activity data were fitted to the Hanes–Woolf equation, which follows Equation (1), a rearrangement of the Michaelis–Menten equation, in which the ATP/*v* ratio is represented as a function of [ATP].

(1) $$\frac{\left[S\right]}{v}=\frac{1}{V_{max}}\;[S]+\frac{K_{m}}{V_{max}}$$

All tests were performed without ASC and maintaining a fixed concentration of 2 mM ASC, varying the initial substrate concentrations within a range of 2 to 6 mM ATP. To account for the limited range of substrate concentrations, the Hanes–Woolf transformation was chosen to minimize statistical distortion and distribute experimental errors more evenly across the regression analysis.

*V_max_* and *K_m_* values were determined by least-squares linear regression analysis. Specifically, *V_max_* was calculated from the reciprocal of the slope of the resulting lines, and *K_m_* was obtained from the intercept with the *x*-axis (transformed to a positive value). The *R*^2^ value was always higher than 0.80, confirming the linearity of these plots.

### 2.5. Quantitative Evaluation of Free Thiols

Free thiols in mitochondrial suspensions in the absence and in the presence of ASC, NAC and both in combination were determined using Ellman’s colorimetric assay [21]. Freshly prepared ASC, NAC or both in combination were added to the mitochondrial suspensions immediately before the analysis. Ellman’s method is based on the reaction of 5,5′-Dithiobis (2-nitrobenzoic acid) (DTNB) with free sulfhydryl (-SH) producing thionitrobenzoic acid (TNB), whose formation is directly proportional to the amount of accessible thiols. Proteins were precipitated by adding 15% (*w*/*v*) trichloroacetic acid, and the samples were centrifuged at 12,000× *g* for 5 min at 4 °C. After discarding the supernatant, the mitochondrial pellet was gently resuspended using a potter homogenizer. Then, 400 μL of reaction solution containing 0.5 M phosphate buffer (KH_2_PO_4_/K_2_HPO_4_, pH 7.4) and 0.2 mM DTNB was added to each sample and incubated for 20 min at 4 °C. Subsequently, the suspensions were centrifuged again at 12,000× *g* for 5 min at 4 °C. The absorbance of the supernatant was measured at 412 nm, corresponding to the maximum TNB absorption, using spectrophotometer. Mitochondrial free thiol content was quantified by interpolating the absorbance values in a calibration curve generated by known cysteine concentrations as –SH standard.

### 2.6. Mitochondrial Respiration Assay

Mitochondrial respiration was evaluated using an extracellular flux analyzer (Seahorse XF HS Mini Analyzer, Agilent Technologies, Santa Clara, CA, USA), which allows real-time measurement of oxygen consumption rate (OCR). A total of 2 μg of frozen/thawed mitochondria was loaded into each well’s Seahorse plate and centrifuged for 15 min at 20,000 *g* and 4 °C to allow mitochondrial adherence to the well bottom of the Seahorse plate in 25 μL of medium A (290 mM sucrose, 2 mM Hepes, pH 7.2 with Tris) containing 10 mM NADH to evaluate NADH-O_2_ oxidase activity or 10 mM of succinate and 20 nM rotenon to evaluate succinate–O_2_ oxidase activity. Additionally, 1 μM antimycin A (AA), a CIII inhibitor, was used in different wells to obtain non-specific OCR, subtracted from all other OCR values. After centrifugation, 180 μL per well of medium A supplemented with substrate or AA was added to the selected wells. Mitochondrial respiration in the absence and presence of increasing concentrations of ASC, NAC and both in combination was investigated by sequential injections of the considered compound or combination through the Seahorse injection ports. The injection ports of the XFp sensor cartridges were hydrated overnight with XF Calibrator at 37 °C. The next day, the cartridges were loaded with a certain desired concentration of ASC, NAC and their combination. OCR values were recorded after each considered concentration of compound injection. In control wells, medium A was injected. OCR data were analyzed using WAVE software 2.6.4., calculated per well in three independent experiments, and normalized to μg of protein per well.

### 2.7. Statistical Analysis

The data represent the mean ± SD (shown as vertical bars in the figures) of the number of experiments reported in the figure captions. One-way ANOVA followed by Dunnett’s multiple-comparison test was used to compare each treatment group with the untreated control. A *p* value < 0.05 was considered significant. For comparisons between two groups, an unpaired two-tailed Student’s *t*-test was performed. GraphPad Prism statistical software (Version 10.6.1, GraphPad Software Inc., La Jolla, CA, USA) was used for statistical analysis.

## 3. Results

### 3.1. Mitochondrial F-ATPase Activity Assay Results

**Figure 1:**
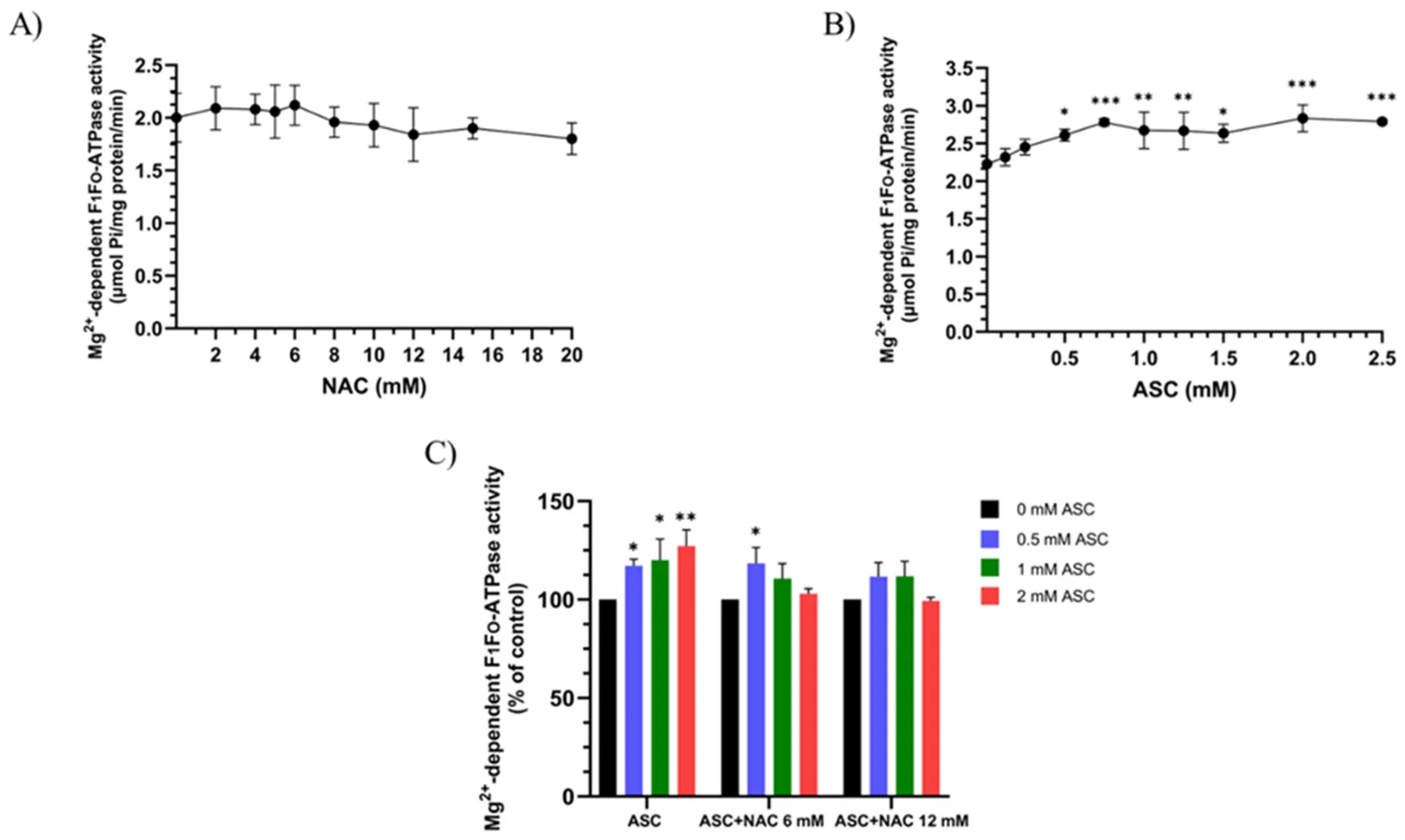
ASC and NAC titration curve on mitochondrial Mg^2+^-dependent F1FO-ATPase at increasing ASC (**A**) and NAC (**B**) concentrations. The effect of combined treatment (**C**) was evaluated in the presence of 0.5 mM (blue bars, 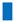), 1 mM (green bars, 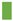) and 2 mM of ASC (red bars, 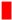) with 6 and 12 mM of NAC. The enzyme activity under combined treatment conditions is expressed as percentage of the control (100%). Data represent the mean ± SD (vertical bars) from three independent experiments carried out on distinct mitochondrial preparations. * (*p* ≤ 0.05), ** (*p* ≤ 0.01), and *** (*p* ≤ 0.001) versus control.

The effect of ASC on Mg^2+^-dependent F_1_F_O_-ATPase was evaluated in the concentration range 0.125–2.5 mM (Figure 1A). ASC significantly increased the Mg^2+^-dependent hydrolytic activity of mitochondrial F_1_F_O_-ATPase in a concentration-dependent manner. In particular, the stimulatory effect became significant at a concentration of 0.5 mM and reached a plateau at concentrations ≥ 0.75 mM with about 20% activation, remaining significantly higher than the control up to 2.5 mM, where a maximal percentage activation (24%) was observed. NAC treatment alone did not significantly modify the Mg^2+^-dependent hydrolytic activity of mitochondrial F_1_F_O_-ATPase over the concentration range tested (2–20 mM) (Figure 1B). Although a slight decrease in enzyme activity was observed from 8 mM onward, the values were not significantly different from the control.

We also investigated the combined treatment of 0.5, 1 and 2 mM ASC in the absence or presence of 6 and 12 mM NAC. The stimulatory effect induced by increasing concentration of ASC alone was progressively reduced in the presence of 6 mM of NAC (Figure 1C). At the highest NAC concentration tested, this effect was more pronounced, with F_1_F_O_-ATPase activity at the corresponding ASC concentrations becoming comparable to control values.

### 3.2. Kinetic Study Results

**Figure 2:**
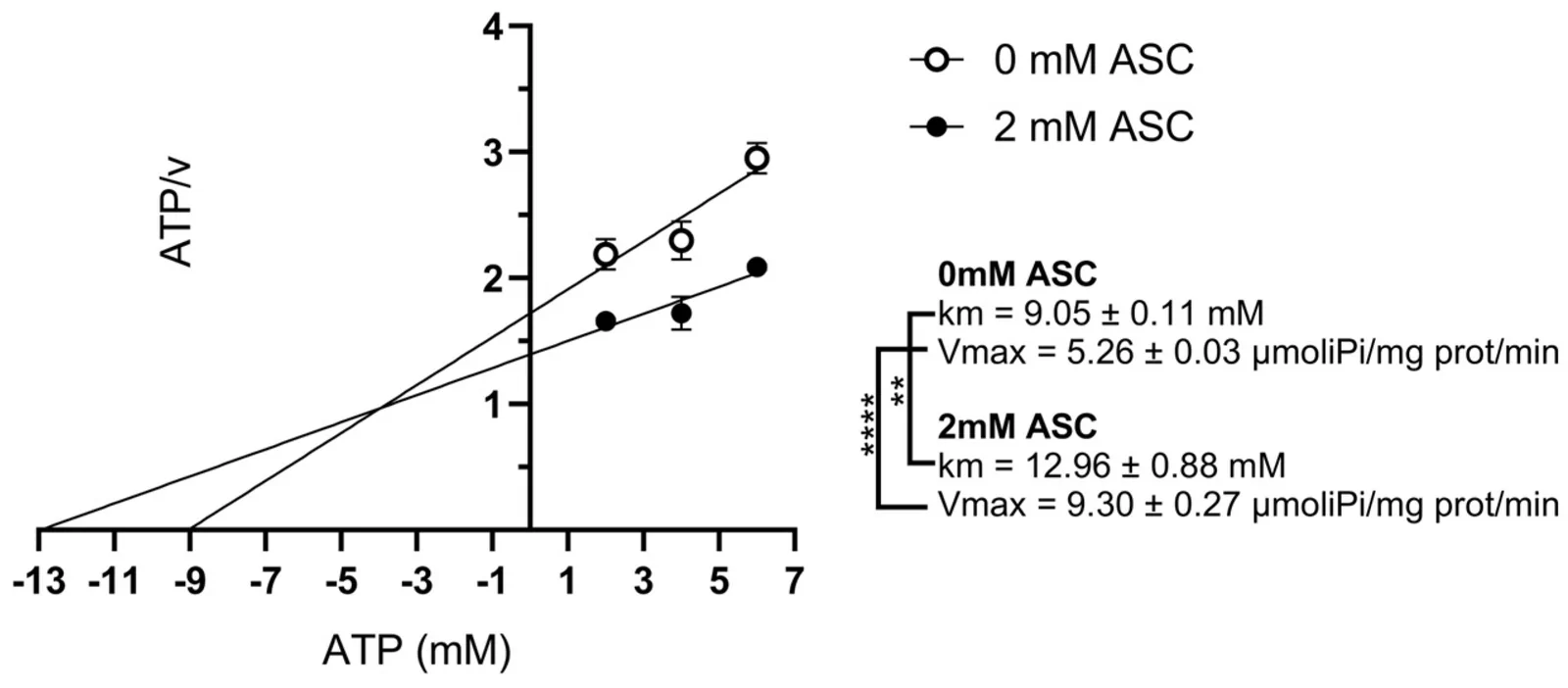
Evaluation of the kinetic mechanism of ASC on Mg^2+^-dependent F_1_F_O_-ATPase. Hanes–Woolf plot of the Mg^2+^-dependent F_1_F_O_-ATPase with respect to ATP substrate in the absence (○) or with 2 mM ASC (●). Values of *K_m_* and *V_max_* are reported for 0 mM ASC and 2 mM ASC conditions, respectively. The addition of 2 mM ASC resulted in a significant increase in both *K_m_* (** *p* = 0.0016) and *V_max_* (**** *p* < 0.0001) compared to the control (unpaired Student’s *t*-test). Each point represents the mean ± SD (vertical bars) of at least three experiments performed on distinct mitochondrial preparations.

To explore the mechanism of ASC activation on the Mg^2+^-dependent F_1_F_O_-ATPase, a Hanes–Woolf plot was constructed using different ATP concentrations (2, 4, and 6 mM) in the absence or presence of 2 mM ASC (Figure 2). Analyzing the activation effect of ASC on the Mg^2+^-dependent F_1_F_O_ ATPase compared to the ATP substrate allows us to hypothesize a mixed uncompetitive activation based on the *K_m_* and *V_max_* values that increase in the presence of 2 mM ASC (Figure 2). In particular, analysis of kinetic parameters showed that the administration of 2 mM ASC determined a statistically significant increase in both *K_m_* (12.96 vs. 9.050; *p* = 0.0016) and *V_max_* (9.300 vs. 5.260; *p* < 0.0001, unpaired Student’s *t*-test).

This indicates that ASC may prefer to bind to the enzyme when it is already complexed with the ATP substrate, forming the ternary enzyme–activator–substrate (EAS) complex, rather than to the free enzyme. Overall, the kinetic data suggest that ASC behaved as a mixed uncompetitive activator.

### 3.3. Results of Quantitative Evaluation of Free Thiols

**Figure 3:**
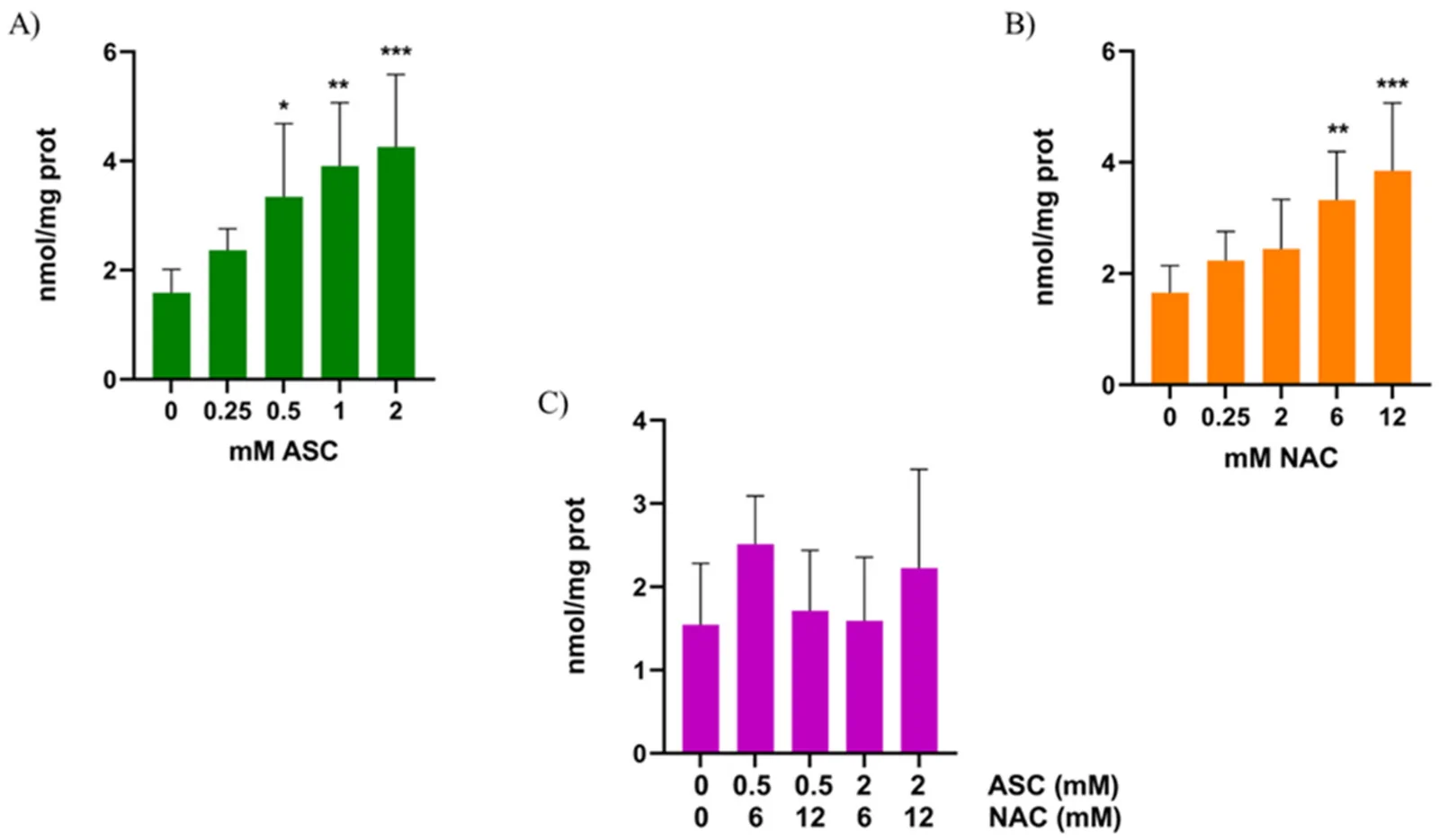
Mitochondrial free thiol (–SH) content in the absence or in the presence of increasing concentrations of ASC (**A**) (green bars, 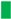), NAC (**B**) (orange bars, 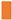) or their combination (**C**) (purple bars, 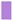). Data are expressed as mean ± SD from four independent experiments carried out on distinct mitochondrial preparations. * (*p* < 0.05), ** (*p* < 0.01), and *** (*p* < 0.001) versus control.

Considering the antioxidant properties of ASC and NAC and their ability to preserve the cellular thiol redox state, their effects on mitochondrial free thiol content were evaluated in swine heart mitochondria incubated in the absence or presence of ASC, NAC and their combination. The free thiol content of mitochondria increased in a concentration-dependent manner following ASC treatment, becoming statistically significant from 0.5 mM onward (Figure 3A). Similarly, NAC significantly increased mitochondrial free thiol levels in a dose-dependent manner (Figure 3B). Conversely, the combined treatment with ASC and NAC (Figure 3C) did not produce a statistically significant increase in free thiol content compared to the action of individual compounds.

### 3.4. Mitochondrial Respiration Assay Results

**Figure 4:**
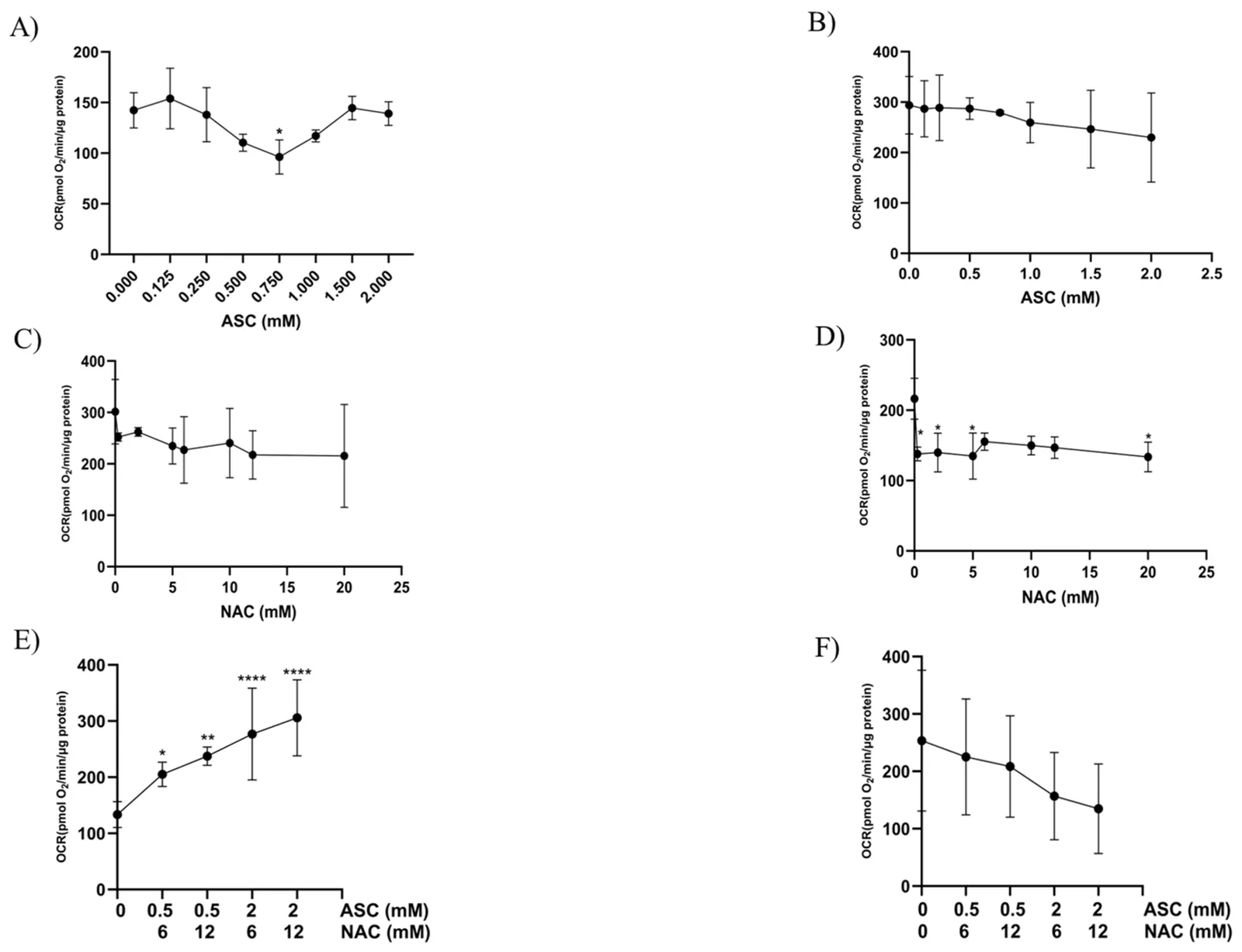
Effect of ASC (**A**,**B**), NAC (**C**,**D**) and their combination (**E**,**F**) on mitochondrial respiration. NADH-O_2_ oxidoreductase activity (**A**,**C**,**E**) and succinate-O_2_ oxidoreductase activity (**B**,**D**,**F**) in the presence of increasing concentrations of compounds treatment. The data represent the mean ± SD (vertical bars) from three independent experiments performed on different mitochondrial preparations. * (*p* ≤ 0.05), ** (*p* < 0.01), and **** (*p* < 0.0001) versus control.

The effects of ASC, NAC and their combination on mitochondrial respiration were evaluated in NADH- or succinate-energized mitochondria. The compounds’ concentrations selected from titration curves of F-ATPase enzyme activity were tested by measuring the oxygen consumption in uncoupled (freeze-thawed) mitochondria in the presence of either NADH or succinate as respiratory substrates. These substrates, respectively, support the NADH-O_2_ oxidoreductase activity (Complex I + Complex III + Complex IV) and succinate-O_2_ oxidoreductase activity (Complex II + Complex III + Complex IV). ASC at a concentration of 0.75 mM caused a significant inhibition of NADH-O_2_ oxidoreductase activity, while at higher concentrations, the OCR value returned to control levels (Figure 4A). When succinate was used as substrate, instead, ASC did not influence oxygen consumption (Figure 4B). Conversely, NAC did not significantly affect mitochondrial respiration driven by NADH (Figure 4C) but significantly inhibited succinate-driven respiration by about 38% at the concentrations of 0.25, 2, 5 and 20 mM (Figure 4D). The combined treatment showed a stimulatory effect of NADH-O_2_ oxidoreductase activity in a concentration-dependent manner, reaching a maximum increase at the highest concentrations tested (Figure 4E). In contrast, on mitochondrial respiration driven by succinate, the combined treatment induced a gradual decrease in OCR, although the effect was not statistically significant (Figure 4F).

## 4. Discussion

The present findings indicate that ASC and NAC, although both classified as antioxidant molecules, exert clearly distinct effects on cardiac mitochondrial bioenergetics. ASC significantly stimulated the Mg^2+^-dependent hydrolytic activity of mitochondrial F_1_F_O_-ATPase in a concentration-dependent manner, with a significant effect beginning at 0.5 mM and reaching a plateau at ≥0.75 mM, whereas NAC alone did not significantly modify ATPase activity over the concentration range tested. Importantly, the ASC-dependent activation was progressively lost in the presence of NAC, and at the highest NAC concentration the activity returned close to control values. From an enzymological perspective, this behaviour suggests that ASC does not act as a generic antioxidant on the enzyme, but rather as a functional modulator of the catalytic cycle of Mg^2+^-dependent F_1_F_O_-ATPase. The kinetic analysis supports this interpretation. Hanes’ analysis, conducted at different ATP concentrations in the absence or presence of ASC, indicated a mixed uncompetitive activation mechanism, supported by the apparent increase in Vmax, together with that of apparent Km, implying that ASC may prefer to interact with the enzyme–substrate complex. Therefore, an activator hypothetically acting on the enzyme–substrate complex could influence the transition between catalytic indwelling states rather than simply increasing substrate binding. In this context, we hypothesize that ASC could favour a conformation of the catalytic sector that facilitates ATP hydrolysis under Mg^2+^-dependent conditions. This interpretation is consistent with the general structural and mechanistic-chemical framework of the F_1_F_O_ ATPase, in which ATP hydrolysis and synthesis are tightly coupled to conformational changes and rotor motion. A useful mechanistic reference is the work on the catalysis of IF_1_-controlled ATP synthase, which highlights how ligand/protein interactions can selectively influence the enzyme’s hydrolytic mode without being equivalent to a simple inhibitor or activator of the active site [22].

A central mechanistic issue is the redox sensitivity of mitochondrial F_1_F_O_-ATPase. The increase in free thiol groups induced by ASC and NAC individually indicates that both compounds can shift mitochondrial proteins toward a more reduced thiol state. ASC increased free thiols in a concentration-dependent manner, becoming significant from 0.5 mM, while NAC also significantly increased thiol availability in a dose-dependent manner. However, this effect was not maintained when ASC and NAC were combined. This result suggests that the two compounds do not simply exert additive antioxidant effects in this mitochondrial system. Although not directly assessed in the present study, we hypothesize that the loss of effect observed with the combined treatment could reflect a redox buffering effect between ASC and NAC, a direct chemical interaction, or mutual quenching, thus reducing the availability of each molecule to interact with protein thiols or redox-sensitive catalytic sites.

This interpretation is consistent with the broader concept that mitochondrial F_1_F_O_-ATPase contains redox-responsive thiol groups whose oxidation or reduction can affect enzyme activity and mitochondrial permeability transition. A directly relevant study showed that dithiol reagents modulate F_1_F_O_-ATPase activity and mPTP formation through cysteine-dependent mechanisms [23]. In that work, phenylarsine oxide increased ATP hydrolysis, while dibromobimane inhibited the enzyme, supporting the idea that the functional outcome depends on the specific cysteine pair or thiol state modified rather than on a generic oxidizing or reducing condition.

The fact that NAC increases reduced thiols but does not activate Mg^2+^-dependent F_1_F_O_-ATPase is particularly informative. It indicates that increasing the global pool of reduced mitochondrial thiols is not sufficient to stimulate the hydrolytic activity of the enzyme. Therefore, the activation of Mg^2+^-dependent F_1_F_O_-ATPase by ASC is unlikely to be explained solely by a generalized increase in mitochondrial free thiols. Together with previous evidence demonstrating the redox sensitivity of F_1_F_O_-ATPase [23], these findings are consistent with a possible involvement of thiol-dependent regulatory mechanisms, despite the current work not identifying the specific cysteine residues or protein targets involved. NAC may preserve thiols in a reduced state without promoting the conformational/catalytic transition required for activation. This distinction is important when interpreting antioxidant effects in enzymatic systems: antioxidant capacity does not necessarily predict directionality of enzyme modulation. For NAC pharmacology and thiol-redox biology, a useful recent reference is [24] for clinical and biochemical background on NAC; for a broader and more current thiol-redox interpretation, see [25].

The loss of ASC-dependent F_1_F_O_-ATPase activation in the combined ASC + NAC condition provides an additional mechanistic clue. If ASC preferentially stabilizes the enzyme–substrate–activator complex, the presence of NAC may interfere either with ASC availability, ASC oxidation state, or ASC interaction with the enzyme. This could occur through chemical interaction between the two antioxidants, through altered redox poise, or through competition at redox-sensitive protein microenvironments. The disappearance of the thiol-increasing effect under combined treatment supports the idea that ASC and NAC are not acting independently in this system. Rather than producing stronger reducing conditions, the combination appears to neutralize the biochemical effect observed with each compound alone. The respiratory data further support a substrate-dependent action of ASC and NAC on mitochondrial electron transfer. ASC transiently inhibited NADH-O_2_ oxidoreductase activity at 0.75 mM, but this inhibition disappeared at higher concentrations, with oxygen consumption returning to control values. Conversely, ASC did not significantly affect succinate-O_2_ oxidoreductase activity. NAC showed the opposite profile: it did not significantly affect NADH-driven respiration, but significantly inhibited succinate-driven respiration by approximately 38% at selected concentrations. In combination, ASC and NAC stimulated NADH-O_2_ oxidoreductase activity in a concentration-dependent manner, while succinate-O_2_ oxidoreductase activity remained statistically unchanged. These observations indicate that ASC and NAC modulate mitochondrial respiration through pathway-specific mechanisms. The selective ASC effect on NADH-supported respiration suggests a redox-sensitive interaction with the Complex I-linked respiratory chain segment, either at Complex I itself, at the downstream quinone/Complex III segment, or at redox equilibria influencing NADH-dependent electron flux. Complex I is highly redox-sensitive and contains multiple redox-active prosthetic groups and cysteine residues that can behave as regulatory nodes [26]. Given that ASC + NAC selectively enhances NADH-O_2_ oxidoreductase activity, future studies should assess the combined effect of ASC, NAC, and CoQ_10_ to determine whether CoQ_10_, as a mobile electron carrier and redox-active lipid antioxidant, can further support Complex I-linked electron flux and mitochondrial respiratory efficiency [27,28].

The NAC-dependent inhibition of succinate-driven respiration suggests that NAC may affect the Complex II-linked pathway. Since ASC did not reproduce this effect, the inhibition cannot be attributed simply to a more reduced mitochondrial environment. It may instead reflect a compound-specific action of NAC, possibly related to thiol exchange chemistry, acetylation-related effects, or altered redox interactions within the succinate-supported respiratory chain [25]. The combined ASC + NAC stimulation of NADH-O_2_ oxidoreductase activity, despite the loss of ASC-dependent ATPase activation and the loss of additive thiol protection, is an important result. It suggests that the interaction between ASC and NAC generates a biochemical condition that is not equivalent to either molecule alone. One possibility is that the combination modifies the redox buffering capacity of the mitochondrial preparation in a way that favours NADH-linked electron flow while preventing ASC from interacting productively with F_1_F_O_-ATPase. This would explain why the combined treatment abolishes the ASC effect on ATP hydrolysis but stimulates NADH-supported respiration. It also reinforces the concept that F_1_F_O_-ATPase modulation and respiratory chain modulation are coupled but not identical readouts of mitochondrial redox control.

## 5. Conclusions

On balance, ASC and NAC exert distinct, non-additive effects on isolated swine heart mitochondria. ASC acts as a redox-dependent activator of Mg^2+^-dependent F_1_F_O_-ATPase hydrolysis, whereas NAC alone does not significantly affect enzyme activity, despite increasing mitochondrial reduced thiol availability. Kinetic analysis indicates that ASC behaves as a mixed uncompetitive activator, hypothetically compatible with a preferential stabilization of the enzyme–substrate–ASC ternary complex. This suggests that ASC may modulate a catalytically engaged state of F_1_F_O_-ATPase rather than acting only as a generic antioxidant.

When ASC and NAC are combined, the ASC-dependent activation of F_1_F_O_-ATPase is abolished, and the increase in free thiol content observed with each compound alone is lost. This likely reflects possible chemical or redox interaction between the two antioxidants, reducing their individual effects on mitochondrial protein thiols or redox-sensitive catalytic sites.

Respiratory assays further show substrate-dependent effects. ASC mainly affects NADH-linked respiration without significantly altering succinate-O_2_ oxidoreductase activity, whereas NAC inhibits succinate-supported respiration without affecting NADH-O_2_ oxidoreductase activity. Combined ASC and NAC stimulate NADH-O_2_ oxidoreductase activity while leaving succinate-driven respiration largely unchanged.

Overall, these findings show that antioxidant molecules can differentially regulate mitochondrial enzymes and respiratory pathways, supporting a rational evaluation of antioxidant combinations in mitochondrial-targeted strategies.

## Data Availability

The data that support the findings of this study are openly available on AMSActa Institutional Research Repository by AlmaDL University of Bologna Digital Library https://doi.org/10.6092/unibo/amsacta/9069.

## Abbreviations

The following abbreviations are used in this manuscript:

AA	Antimycin A
ASC	Sodium ascorbate
ATP	Adenosine triphosphate
ETC	Electron transport chain
GSH	Glutathione
*K_m_*	Michaelis–Menten constant
mtDNA	Mitochondrial DNA
NAC	N-acetyl-L-cysteine
NADH	Nicotinamide adenine dinucleotide (reduced form)
OCR	Oxygen consumption rate
OXPHOS	Oxidative phosphorylation
ROS	Reactive oxygen species
*R*^2^	Coefficient of determination
*V_max_*	Maximum reaction velocity

## References

[1] Nolfi-Donegan D., Braganza A., Shiva S. (2020). Mitochondrial Electron Transport Chain: Oxidative Phosphorylation, Oxidant Production, and Methods of Measurement. Redox Biol..

[2] Cogliati S., Cabrera-Alarcón J.L., Enriquez J.A. (2021). Regulation and Functional Role of the Electron Transport Chain Supercomplexes. Biochem. Soc. Trans..

[3] Javadov S., Kozlov A.V., Camara A.K.S. (2020). Mitochondria in Health and Diseases. Cells.

[4] Hong Y., Boiti A., Vallone D., Foulkes N.S. (2024). Reactive Oxygen Species Signaling and Oxidative Stress: Transcriptional Regulation and Evolution. Antioxidants.

[5] Mailloux R.J. (2020). An Update on Mitochondrial Reactive Oxygen Species Production. Antioxidants.

[6] Singh G., Pachouri U.C., Khaidem D.C., Kundu A., Chopra C., Singh P. (2015). Mitochondrial DNA Damage and Diseases. F1000Research.

[7] Xu X., Pang Y., Fan X. (2025). Mitochondria in Oxidative Stress, Inflammation and Aging: From Mechanisms to Therapeutic Advances. Signal Transduct. Target. Ther..

[8] Gonzalez M.J., Miranda-Massari J.R., Olalde J., Ostojic S.M. (2023). Chapter 9—Vitamin C and Mitochondrial Function in Health and Exercise. Molecular Nutrition and Mitochondria.

[9] Gęgotek A., Skrzydlewska E. (2022). Antioxidative and Anti-Inflammatory Activity of Ascorbic Acid. Antioxidants.

[10] Guan Y., Dai P., Wang H. (2020). Effects of Vitamin C Supplementation on Essential Hypertension. Medicine.

[11] Berretta M., Quagliariello V., Maurea N., Di Francia R., Sharifi S., Facchini G., Rinaldi L., Piezzo M., Manuela C., Nunnari G. (2020). Multiple Effects of Ascorbic Acid against Chronic Diseases: Updated Evidence from Preclinical and Clinical Studies. Antioxidants.

[12] Tenório M.C.d.S., Graciliano N.G., Moura F.A., de Oliveira A.C.M., Goulart M.O.F. (2021). N-Acetylcysteine (NAC): Impacts on Human Health. Antioxidants.

[13] Mokhtari V., Afsharian P., Shahhoseini M., Kalantar S.M., Moini A. (2017). A Review on Various Uses of N-Acetyl Cysteine. Cell J..

[14] Samuni Y., Goldstein S., Dean O.M., Berk M. (2013). The Chemistry and Biological Activities of N-Acetylcysteine. Biochim. Biophys. Acta (BBA)–Gen. Subj..

[15] Wright D.J., Renoir T., Smith Z.M., Frazier A.E., Francis P.S., Thorburn D.R., McGee S.L., Hannan A.J., Gray L.J. (2015). N-Acetylcysteine Improves Mitochondrial Function and Ameliorates Behavioral Deficits in the R6/1 Mouse Model of Huntington’s Disease. Transl. Psychiatry.

[16] Zheng J., Zhao L., Liu Y., Chen M., Guo X., Wang J. (2024). N-Acetylcysteine, a Small Molecule Scavenger of Reactive Oxygen Species, Alleviates Cardiomyocyte Damage by Regulating OPA1-Mediated Mitochondrial Quality Control and Apoptosis in Response to Oxidative Stress. J. Thorac. Dis..

[17] Zhou J., Terluk M.R., Orchard P.J., Cloyd J.C., Kartha R.V. (2021). N-Acetylcysteine Reverses the Mitochondrial Dysfunction Induced by Very Long-Chain Fatty Acids in Murine Oligodendrocyte Model of Adrenoleukodystrophy. Biomedicines.

[18] Bradford M.M. (1976). A Rapid and Sensitive Method for the Quantitation of Microgram Quantities of Protein Utilizing the Principle of Protein-Dye Binding. Anal. Biochem..

[19] Ventrella V., Nesci S., Trombetti F., Bandiera P., Pirini M., Borgatti A.R., Pagliarani A. (2011). Tributyltin Inhibits the Oligomycin-Sensitive Mg-ATPase Activity in Mytilus Galloprovincialis Digestive Gland Mitochondria. Comp. Biochem. Physiol. Part C Toxicol. Pharmacol..

[20] Algieri C., Trombetti F., Pagliarani A., Ventrella V., Bernardini C., Fabbri M., Forni M., Nesci S. (2019). Mitochondrial Ca^2+^-Activated F_1_F_O_-ATPase Hydrolyzes ATP and Promotes the Permeability Transition Pore. Ann. N. Y. Acad. Sci..

[21] Ellman G.L. (1959). Tissue Sulfhydryl Groups. Arch. Biochem. Biophys..

[22] Kobayashi R., Ueno H., Okazaki K., Noji H. (2023). Molecular Mechanism on Forcible Ejection of ATPase Inhibitory Factor 1 from Mitochondrial ATP Synthase. Nat. Commun..

[23] Algieri C., Trombetti F., Pagliarani A., Ventrella V., Nesci S. (2021). The Mitochondrial F_1_F_O_-ATPase Exploits the Dithiol Redox State to Modulate the Permeability Transition Pore. Arch. Biochem. Biophys..

[24] Schwalfenberg G.K. (2021). N-Acetylcysteine: A Review of Clinical Usefulness (an Old Drug with New Tricks). J. Nutr. Metab..

[25] Qu H.Q., Kao C., Hakonarson H. (2026). Redefining the Role of the Thiol-Based Agent N-Acetylcysteine in Human Health and Disease and Elucidating Potential Advantages of Its Amide Derivative. RSC Med. Chem..

[26] Chen R., Tabatabaei Dakhili S.A., Gerulskis R., Zhao Y.-Y., Lockhart S., Tonoyan L., Siraki A.G., Huang G., Kinnaird A., Freed D.H. (2025). Cysteine Oxidation of a Redox Hub within Complex I Can Facilitate Electron Transport Chain Supercomplex Formation. J. Biol. Chem..

[27] Guarás A., Perales-Clemente E., Calvo E., Acín-Pérez R., Loureiro-Lopez M., Pujol C., Martínez-Carrascoso I., Nuñez E., García-Marqués F., Rodríguez-Hernández M.A. (2016). The CoQH2/CoQ Ratio Serves as a Sensor of Respiratory Chain Efficiency. Cell Rep..

[28] Nesci S., Algieri C., Trombetti F., Fabbri M., Lenaz G. (2023). Two Separate Pathways Underlie NADH and Succinate Oxidation in Swine Heart Mitochondria: Kinetic Evidence on the Mobile Electron Carriers. Biochim. Biophys. Acta (BBA)–Bioenerg..

